# CYC31, A Natural Bromophenol PTP1B Inhibitor, Activates Insulin Signaling and Improves Long Chain-Fatty Acid Oxidation in C2C12 Myotubes

**DOI:** 10.3390/md18050267

**Published:** 2020-05-19

**Authors:** Jiao Luo, Yufei Hou, Mengyue Xie, Wanli Ma, Dayong Shi, Bo Jiang

**Affiliations:** 1 School of Public Health, Qingdao University, Qingdao 266071, China; luojiao2012@163.com (J.L.); yifho1007@163.com (Y.H.); xiemengyue11@163.com (M.X.); wanli_ma001@163.com (W.M.); 2 Key Laboratory of Experimental Marine Biology, Institute of Oceanology, Chinese Academy of Sciences, 7 Nanhai Road, Qingdao 266071, China; 3 State Key Laboratory of Microbial Technology, Shandong University, Jinan 250100, China; 4 Center for Ocean Mega-Science, Chinese Academy of Sciences, 7 Nanhai Road, Qingdao 266071, China

**Keywords:** CYC31, PTP1B inhibitor, insulin resistance, fatty acid oxidization, C2C12

## Abstract

3-bromo-4,5-Bis(2,3-dibromo-4,5-dihydroxybenzyl)-1,2-benzenediol (CYC31) is a bromophenol protein tyrosine phosphatase 1B (PTP1B) inhibitor isolated from the red alga *Rhodomela confervoides*. Here, the effect of CYC31 on the insulin signaling and fatty-acid-induced disorders in C2C12 myotubes was investigated. Molecular docking assay showed that CYC31 was embedded into the catalytic pocket of PTP1B. A cellular study found that CYC31 increased the activity of insulin signaling and promoted 2-NBDG uptake through GLUT4 translocation in C2C12 myotubes. Further studies showed that CYC31 ameliorated palmitate-induced insulin resistance in C2C12 myotubes. Moreover, CYC31 treatment significantly increased the mRNA expression of carnitine palmitoyltransferase 1B (*CPT-1B*) and fatty acid binding protein 3 (*FABP3*), which was tightly linked with fatty acid oxidation. These findings suggested that CYC31 could prevent palmitate-induce insulin resistance and could improve fatty acid oxidation through PTP1B inhibition.

## 1. Introduction

Insulin resistance is a common pathological condition in obesity and type 2 diabetes, which is characterized by an impaired response to insulin in peripheral tissues [1,2]. Up to 75% of insulin-dependent glucose is handled by skeletal muscle cells, and muscle plays a central role in whole-body insulin resistance [3]. It is well established that the high incidence rate of insulin resistance is, to a large extent, due to the epidemic of obesity [4]. Although the mechanisms underlying the relationship between obesity and skeletal muscle insulin resistance remain controversial, the prevailing theory links insulin resistance to the increasing circulating free fatty acids (FFA) [5]. It is reported that elevated plasma FFA levels in obesity subjects caused insulin resistance in peripheral tissues [6]. The theory also demonstrates that mitochondria are critical players in the obesity-linked insulin resistance [7,8]. It is believed that the decrease of mitochondrial fatty acid oxidation caused by mitochondrial dysfunction contributes to insulin resistance development in skeletal muscle [9] because the decrease of fatty acid oxidation leads to the increase of intracellular fatty acyl-CoA and diacylglycerol levels, which attenuate insulin signaling.

Protein tyrosine phosphatase 1B (PTP1B) catalyzes the dephosphorylation of insulin receptor (IR); thus, it is a negative regulator of insulin signaling [10,11,12]. Whole-body PTP1B-knockout mice are resistant to high fat-induced obesity with enhanced insulin sensitivity and do not develop T2DM [13,14]. The study of tissue-specific PTP1B knockout mice demonstrates that PTP1B is a major modulator of insulin sensitivity, glucose homeostasis, and lipid metabolism [15,16,17]. It is also reported that small-molecule PTP1B inhibitors ameliorate insulin resistance both in plamitate-treated cells and high fat diet-induced obesity mice [18,19,20].

**Figure 1:**
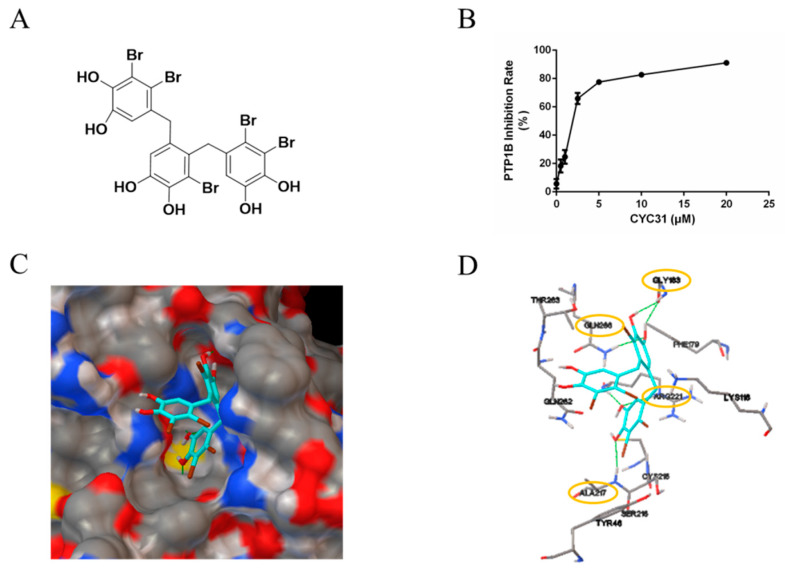
PTP1B inhibitory activity of CYC31: (**A**) Chemical structure of CYC31. (**B**) Concentration-dependent inhibition of PTP1B activity by CYC31. By using p-nitrophenyl phosphate (p-NPP) as substrates, recombinant PTP1B was incubated with different dosages of CYC31 (0.5, 1, 2.5, 5, 10, and 20 μM, respectively) in Tris buffer (pH 7.5) at 37 °C for 30 min, and then, PTP1B activity was measured by detecting the absorbance at 405 nM. (**C**) Surface representation and (**D**) stick representation of predicted binding models of PTP1B (3QKP) with CYC31. CYC31 was colored light blue. Oxygen, sulfur, carbon, nitrogen, and bromine atoms are colored red, yellow, gray, blue, and green, respectively. The hydrogen bonds are displayed as green lines.

The marine environment is a rich pool of novel bioactive natural products [21]. Previously, we found 3-bromo-4,5-bis(2,3-dibromo-4,5-dihydroxybenzyl)-1,2-benzenediol (CYC31, also called BDB; chemical structure was shown in Figure 1A), a natural bromophenol compound isolated from the red alga *Rhodomela confervoides*, displayed potent PTP1B inhibition activity (IC_50_ 1.7 µM) [22,23]. Based on previous research, we hypothesized that PTP1B inhibition by CYC31 might improve the fatty acid-induced functional disorders in C2C12 myotubes. Therefore, in the present study, we aimed to elucidate the effects of CYC31 on the insulin signaling pathway, fatty acid-induced insulin resistance, and fatty acid oxidation signaling in C2C12 myotubes.

## 2. Results

### 2.1. CYC31 Embeds Into the Catalytic Pocket of PTP1B

First, the in vitro enzymatic experiment was carried out to confirm the PTP1B inhibitory effect of CYC31. As shown in Figure 1B, PTP1B was significantly inactivated by CYC31 in a concentration-dependent manner.

Binding of CYC31 to PTP1B was then investigated by molecular docking. The original ligand binds to the active site, and we imported CYC31 after removing the original ligand. As shown in Figure 1C, the compound positioned itself easily in the catalytic site of PTP1B (PDB code 3QKP). One diphenol group at the bottom of the active site formed an extensive hydrogen bonding network with N-H of residues Ala217 and Arg221 (Figure 1C,D). This group in part mimics the interactions of phosphoryl group in pTyr1162 (insulin receptor kinase) with the active site in PTP1B. The hydroxyl group on middle phenyl ring formed three hydrogen bonds with residues Gly183 and Gln266 (Figure 1D). The third group stretched outside of the binding pocket, and no evident interaction was observed with residues around the active site. 

### 2.2. CYC31 Increases the Activity of Insulin Signaling in C2C12 Myotubes

**Figure 2:**
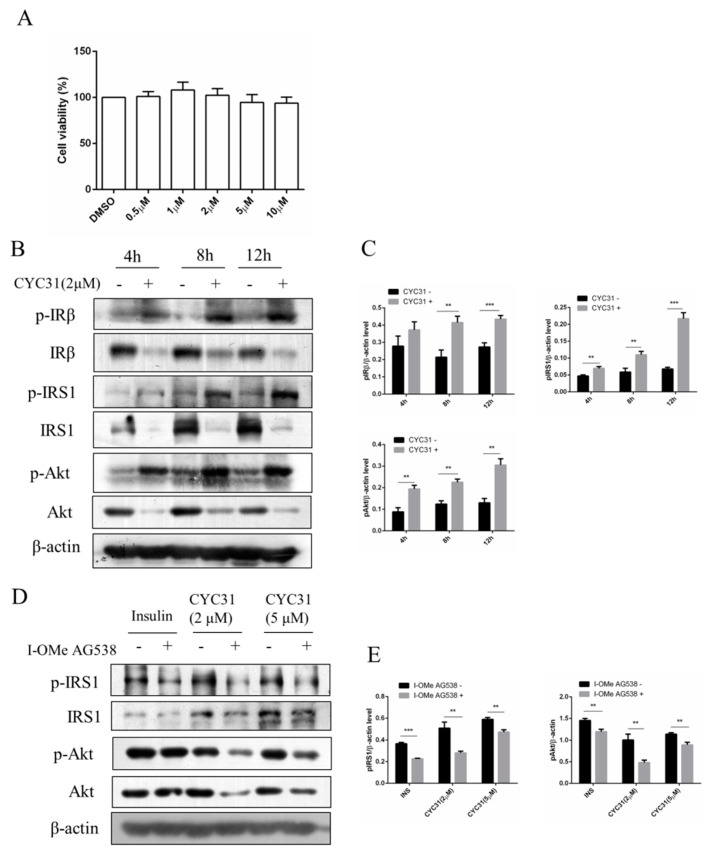
Increased insulin signaling by CYC31: (**A**) The cytotoxicity of CYC31 in C2C12 skeletal cells. C2C12 myotubes were treated with different dosages of CYC31 for 24 h, and the cell viability was measured by MTT assay (**B**) Time-dependent activation of insulin signaling by CYC31: Serum-starved C2C12 myotubes were treated with 2 µM CYC31 for 4–12 h. The phosphorylation of IRS1, IRβ, and Akt, and corresponding total proteins were detected by immunoblotting (**C**) Relative phosphorylation levels of IRS1, IRβ, and Akt. Band density was measured by Image J software and normalized to β-Actin. (**D**) Effect of I-OMe AG538 (IR kinase inhibitor) on CYC31-treated C2C12 skeletal cells. Serum-starved C2C12 myotubes were pretreated with/without 50 µM I-OMe AG538 for 3 h and then exposed to CYC31 for another 8 h. Then, the phosphorylation of IRS1, IRβ, and Akt were detected by western blotting. (**E**) Relative phosphorylation levels of IRS1 and Akt. Band density was measured by Image J software and normalized to β-Actin. Data was shown as mean ± SD values (n = 3), ** *p* < 0.01, *** *p* < 0.001.

We first examined the cytotoxicity of CYC31 on C2C12 myotubes. As shown in Figure 2A, varying concentrations (0–10 μM) of CYC31 had no effect on the viability of C2C12 myotubes after 24 h treatment. Accordingly, further studies on the cellular activities of CYC31 were conducted at a concentration of less than 10 μM.

Because PTP1B is a negative regulator of insulin signaling, we examined the effects of CYC31 on the insulin signaling pathway in C2C12 myotubes. As expected, CYC31 increased the phosphorylation of IR at Tyr1146, one of the major auto phosphorylation sites (Figure 2B,C). Insulin signaling was also examined with Tyr632 phosphorylation of IRS-1 protein and Ser473 phosphorylation of Akt (Figure 2B,C). Both signals were increased in the CYC31-treated cells, suggesting a molecular increase of insulin signaling.

Moreover, pretreatment C2C12 myotubes with IR kinase inhibitor I-OMe AG538 significantly blocked the phosphorylation of IRS-1 and Akt stimulated by CYC31 (Figure 2D,E). This result indicates that CYC31 activates the insulin signal cascade via the indirect autophosphorylation of insulin receptor.

### 2.3. CYC31 Enhances the Glycogen Synthase Signaling

**Figure 3:**
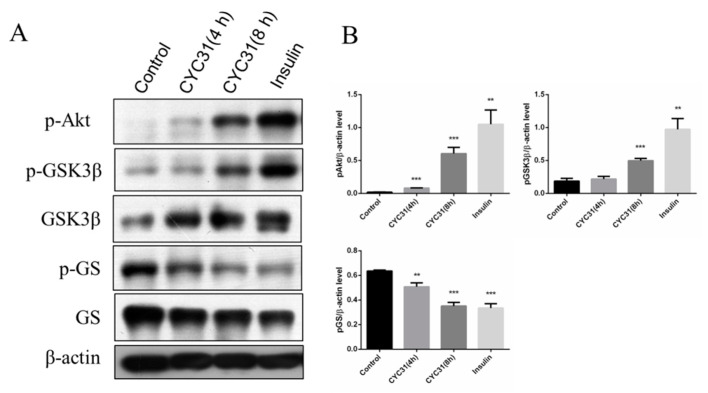
CYC31 activates the glycogen synthesis signaling in C2C12 myotubes: (**A**) Serum-starved C2C12 myotubes were treated with 2 µM CYC31 for 4 h and 8 h. For the insulin-treated group, cells were incubated with 10 nM insulin for 5 min. Then, p-GSK3β, p-GS, and p-Akt were detected by western blotting (**B**) The expression levels of p-Akt, p-GSK3β and p-GS: Band density was measured by Image J software and normalized to β-Actin. Data was shown as mean ± SD values (n = 3), ** *p* < 0.01, *** *p* < 0.001 compared with the DMSO-treated group.

Glycogen synthesis is an important downstream event of insulin signaling pathway. Therefore, we next evaluated the expression of two well-known enzymes involved in muscle glycogen synthesis, glycogen synthase (GS), a rate-limiting enzyme, and GSK3, which dephosphorylates and activates glycogen synthase. CYC31 treatment increased the phosphorylation levels of GSK3β at Ser9 (Figure 3A,B). The phosphorylation of glycogen synthase, the direct target of GSK3, was suppressed in C2C12 cells (Figure 3A,B), indicating that CYC31 enhances the signaling of insulin synthesis.

### 2.4. CYC31 Promotes Glucose Uptake Via GLUT4 Translocation 

**Figure 4:**
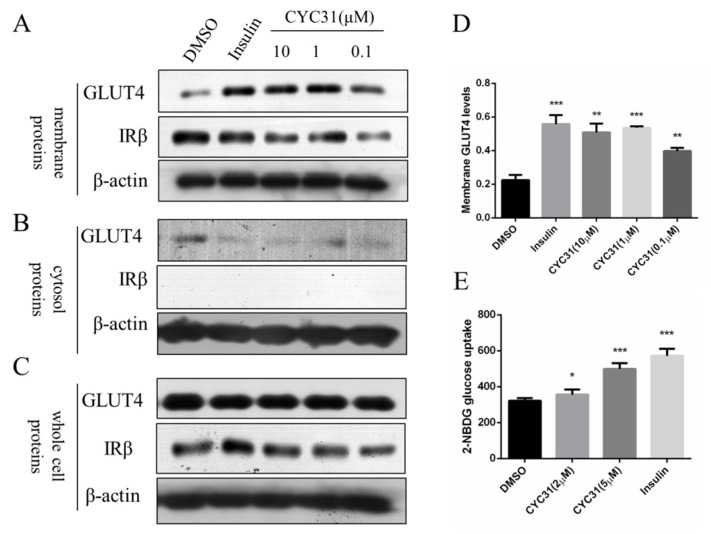
Effect of CYC31 on GLUT4 translocation: (**A**–**C**) The protein level of GLUT4 in the plasma membrane (**A**), cytosol fractions (**B**), and whole cell lysates (**C**) C2C12 myotubes were serum-starved overnight and treated with indicated concentrations of CYC31 for 8 h or 10 nM insulin for 5 min in serum-deprived DMEM; then, cells were lysed and membrane proteins and cytosol fractions were extracted using the Membrane and Cytosol Protein Extraction Kit (Beyotime, Shanghai, China). The protein level of GLUT4 was detected by immunoblotting. (**D**) Relative expression level of GLUT4 on cell membrane after CYC31 treatment: Band density was measured by Image J software and normalized to β-actin. Data was shown as mean ± SD values (n = 3), ** *p* < 0.01, *** *p* < 0.001 compared with the DMSO-treated group (**E**) CYC31 promotes glucose uptake in C2C12 myotubes. C2C12 myotubes were treated with the indicated concentrations of CYC31 for 8 h or 10 nM insulin for 5 min in serum-free DMEM; then, 200 μM 2-NBDG was added for 20 min in glucose-free DMEM. The fluorescence intensity was determined at ex/em 465/540 nm using a microplate fluorometer. The results shown are means ± SD (n = 5). * *p* < 0.05, *** *p* < 0.001 versus the DMSO-treated control group.

We next assessed whether CYC3-increased insulin signaling could promote glucose uptake through GLUT4 translocation to the plasma membrane. C2C12 myotubes were exposed to CYC31 for 8 h and the GLUT4 levels in the fractioned plasma membrane were determined by immunoblotting. IRβ protein was used as the marker of membrane fractions. The result showed that CYC31 treatment increased GLUT4 levels in the plasma membrane (Figure 4A,D) while the expression levels of total GLUT4 in whole cell lysates were not altered (Figure 4C).

To confirm whether CYC31 could enhance glucose uptake, we tested the cellular uptake of 2-NBDG, a fluorescent D-glucose derivate. In agreement with the insulin signaling activation and the GLUT4 translocation, treatment with CYC31 significantly promoted the cellular uptake of 2-NBDG uptake in C2C12 myotubes (Figure 4E).

### 2.5. CYC31 Prevents Palmitate-Induced Insulin Resistance

**Figure 5:**
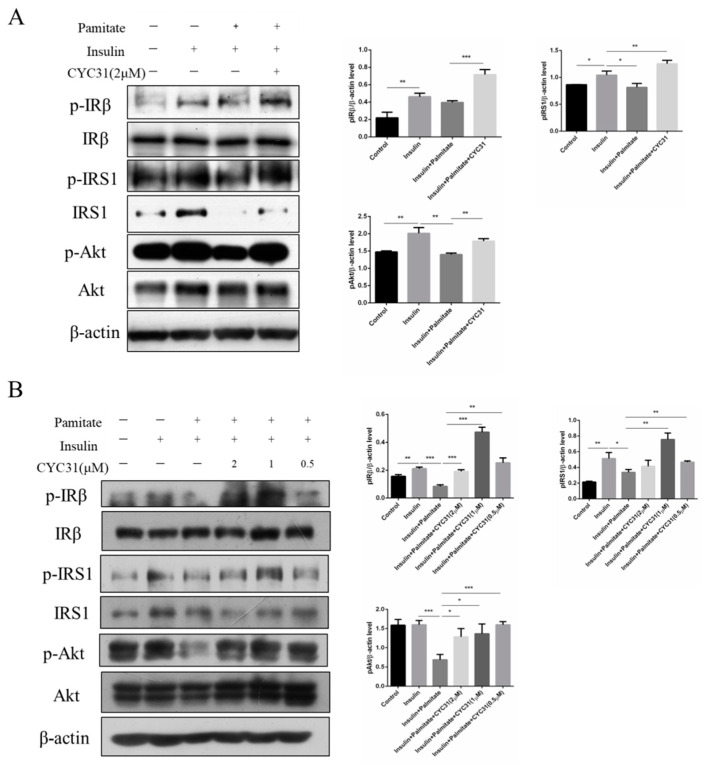
Effect of CYC31 on palmitate-induced insulin resistance: CYC31 ameliorates insulin resistance in C2C12 myotubes (**A**) and HepG2 cells (**B**). Palmitate stock solutions of 100 mM were prepared in 0.1 N NaOH. Before application to the cells, palmitate was conjugated to bovine serum albumin by diluting the palmitate solution with medium containing 1% (w/v) fatty acid-free BSA. Solutions were filter-sterilized before addition to the cells; 0.5 mM palmitate was used to induce insulin resistance in C2C12 and HepG2 cells. Briefly, cells were pre-incubated with CYC31 for 1 h, and then, CYC31-treated cells were exposed to 0.5 mM palmitate for 24 h and starved in FBS-free DMEM (containing compound and palmitate) for another 6 h. Then, cells were incubated with fresh DMEM containing 10 nM insulin for 5 min. The phosphorylation levels of IRβ, IRS1, and Akt and relative total proteins were determined by immunoblotting.

Next, we examined the activity of insulin signaling pathway in the presence of palmitate by phosphorylation of IR, IRS-1, and Akt. As shown in Figure 5A, palmitate treatment induced a significant decrease in the phosphorylation level of IR, IRS-1, and Akt, indicating an insulin resistance state in C2C12 myotubes. In contrast, CYC31 reversed the palmitate-induced insulin resistance by increasing the phosphorylation level of IR, IRS-1, and Akt (Figure 5A). Same results were observed in palmitate-treated HepG2 cells (Figure 5B).

### 2.6. CYC31 Increases the Fatty Acid Oxidation Signal in Palmitate-Exposed C2C12 Myotubes

**Figure 6:**
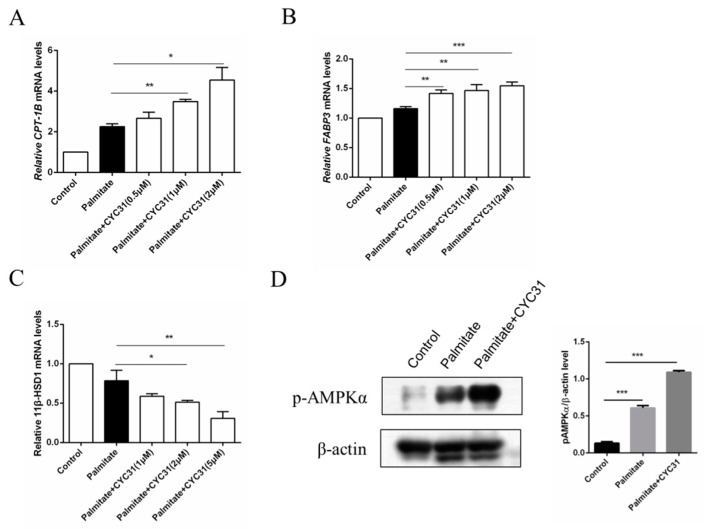
Effect of CYC31 on the long-chain fatty acid oxidation signaling in palmitate-treated C2C12 myotubes: Analysis of the mRNA levels of CPT-1B (**A**), FABP3 (**B**), and 11β-HSD1 (**C**) C2C12 cells were incubated for 24 h in the presence or absence of CYC31 and 0.5 mM palmitate-treated cells (PA). Total RNA was extracted and analyzed by RT-qPCR. * *p* < 0.05, ** *p* < 0.01, *** *p* < 0.001, versus palmitate-treated cells. (**D**) Phosphorylation levels of AMP-activated protein kinase (AMPK) after 2 µM CYC31 treatment. Total protein extracts from C2C12 myotubes were analyzed with phospho-AMPKα(Thr172) antibodies by western blot. PA, palmitate-treated cells.

Increased fatty acid oxidation may attenuate the consequent fatty acid-induced insulin resistance in skeletal muscle cells. Thus, we next evaluated the effect of CYC31 on the expression of genes involved in fatty acid oxidation. Carnitine palmitoyl-transferase-1 gene (CPT-1) is the rate-limiting enzyme of long-chain fatty acid oxidation. Compared with the control cells, palmitate exposure resulted in a 2.2-fold increase in *CPT-1B* mRNA levels (*p* < 0.0017 CYC31 treatment dose-dependently increased *CPT-1B* mRNA levels (Figure 6A). Furthermore, we evaluated the expression of another two well-known genes involved in fatty acid oxidation, including *FABP3* and *11β-HSD1*. Similarly, *FABP3* mRNA levels were increased after PTP1B inhibition by CYC31 (Figure 6B), while *11β-HSD1* mRNA levels were decreased in a dose-dependent manner (Figure 6C). These results suggest that CYC31 promotes the fatty acid oxidation in palmitate-exposed C2C12 myotubes.

It is well established that AMP-activated protein kinase (AMPK) activation increases fatty acid oxidation in skeletal muscle; then, we next tested the phosphorylation levels of AMPK, a key regulator of energy homeostasis. CYC31 treatment significantly increased the phosphorylation level of AMPK at Thr172 in C2C12 myotubes (Figure 6D).

## 3. Discussion

Cellular and animal studies suggest that PTP1B is a negative regulator of insulin and the leptin signaling pathway. Inhibition of PTP1B activity could be an important therapeutic strategy for the treatment of T2DM and obesity [24]. In the present study, we found that CYC31, a PTP1B inhibitor, could ameliorate palmitate-induced insulin resistance and increase fatty acid oxidation signaling.

The ocean possesses unique marine biological resources. Marine bromophenols are a novel kind of chemical widely existing in marine algae. In the current study, CYC31, a natural bromophenol isolated from the red alga *Rhodomela confervoides*, exhibited significant activity inhibition of PTP1B (Figure 1B). A docking study illustrated this inhibition mode in detail, in which CYC31 binds to PTP1B at the catalytic motif through hydrogen bonds at residues Ala217, Arg221, Gly183 and Gln266 (Figure 1C,D). It can be speculated that the CYC31 inhibits PTP1B activity through hydrogen bonds binding to the catalytic site. CYC31 binds to the active catalytic site of PTP1B, which is the same as some bromophenol inhibitors reported [18,25].

CYC31 could activate insulin signaling. In the differentiated C2C12 cells, CYC31 enhanced tyrosine phosphorylation of IRβ in a time-dependent manner (Figure 2B). The enhanced phosphorylation further activated insulin signal pathway by increasing the phosphorylation levels of several downstream factors, including IRS-1 and Akt, which was consistent with the effects of safranal, a PTP1B inhibitor from Saffron [26]. These data suggest that CYC31 could sensitize the insulin pathway in an insulin-independent manner. Moreover, CYC31 treatment did not affect PTP1B protein expression of (data not shown), indicating CYC31 stimulates insulin signaling by cellular PTP1B inactivation rather than PTP1B expression reduction. In addition, CYC31 has its own peculiarities compared with other PTP1B inhibitors. First, CYC31 could activate insulin signal pathway in an insulin-independent manner, while many other PTP1B inhibitors were insulin-dependent, such as CX08005 [19], Norathyriol [20] and JTT-551 [27]. Moreover, the effective concentration of CYC31 was as low as 0.1 µM in C2C12 myocytes. These data suggest that CYC31 has better cell membrane permeability than Norathyriol (1–10 µM) and JTT-551 (3–30 µM).

Insulin resistance of skeletal muscle is mainly manifested in the decrease of glycogen synthesis stimulated by insulin, which in turn leads to the decrease of glucose transport [28]. In the present study, we found that CYC31 effectively prevented palmitate-induced insulin resistance (Figure 5A,B). Furthermore, CYC31 increased the phosphorylation level of GSK3β and reduced the phosphorylation level of GS (Figure 3B), indicating that CYC31 might enhance the glycogen synthesis in C2C12 myotubes. As well-known, GLUT4 plays a key role in the regulation of glucose transport in skeletal muscle. Plasma membrane separation experiment and immunoblotting assay showed that CYC31 strongly increased the plasma membrane GLUT4 levels (Figure 4A). These data indicate that CYC31 promotes the translocation of GLUT4 vesicles from cytoplasm to cell membrane. Figure 4E showed that CYC31 significantly increased the uptake of 2-NBDG in C2C12 myotubes, suggesting that CYC31 can facilitate the entry of glucose inside the cells via promoting GLUT4 translocation. Subsequently, the absorbed glucose is likely to be used for glycogen synthesis and further study should be done to confirm the glycogen content in myotubes. However, there are also limitations of the membrane and cytosol protein extraction technology. Because membrane extract proteins not only include proteins that are in the plasma membrane but also include proteins in the organelles and intracellular vesicles that dock GLUT4, immunofluorescence staining of GLUT4 can be used to confirm the CYC31-induced GLUT4 translocation.

In the diabetic state, insulin action is reduced and fat tissue releases a lot of fatty acids; however, the ability of muscle to oxidise fatty acids is impaired. It is reported that FFA levels were significantly increased in obese subjects when compared with the nonobese individuals, representing 0.714 ± 0.23 and 0.574 ± 0.23 mmol/L, respectively (*p* < 0.0001). Next, we investigated the role of PTP1B as a modulator of fatty acid oxidization, which might be helpful for the amelioration of palmitate-induced insulin resistance. A previous study showed that activation of fatty acid oxidation by CPT-1 overexpression improves lipid-induced insulin resistance in cultured skeletal muscle cells [29] and in mouse skeletal muscle [30]. PTP1B inhibition after CYC31 treatment caused a transcription-mediated increase in the expression of several genes involved in fatty acid oxidization, including *CPT-1B* and *FABP3* (Figure 6A,B). Moreover, CYC31 increased AMPK phosphorylation (Figure 6D), which might also favor palmitate oxidation. AMPK is a fuel-sensing enzyme which can both increase cellular ATP generation (e.g., fatty acid oxidation) and diminish ATP use for less critical processes (e.g., fatty acid synthesis) [31]. In a word, PTP1B inhibition after CYC31 treatment increased the fatty acid oxidation signaling which might further contribute to the improvement of palmitate-induced insulin resistance.

**Figure 7:**
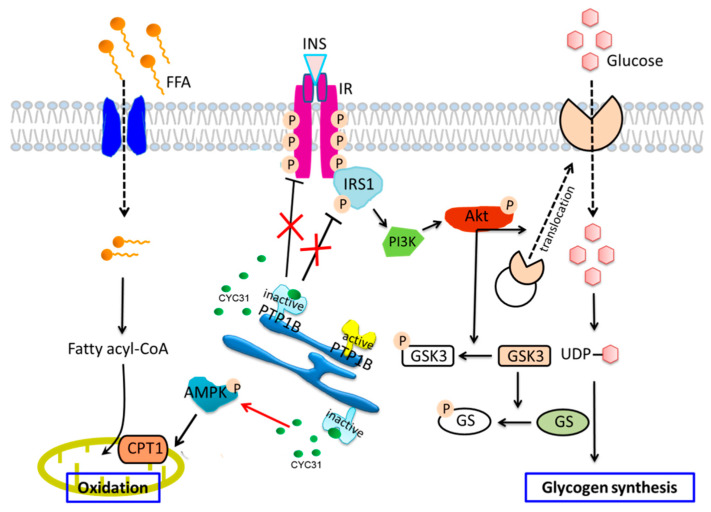
Schematic representation of CYC31 regulation on palmitate-induced insulin resistance and fatty acid oxidation: CYC31 binds to the catalytic domain of PTP1B and inactivates PTP1B, which further recovered palmitate-induced insulin resistance by increasing tyrosine phosphorylation of IRβ and IRS1. Akt was activated by increased upstream signaling of IR and IRS1, thereby promoting GLUT4 translocation to the cell membrane and increasing glucose uptake. Phosphorylated Akt also increases GSK3 phosphorylation and inhibits GS phosphorylation, suggesting potential for enhancing glycogen synthesis. On the other hand, CYC31 enhances AMPK phosphorylation and increases the expression of mitochondrial CPT1, indicating an increased signaling of mitochondrial fatty acid oxidation.

Taken together, on the basis of our findings, we propose that CYC31 activates insulin signaling and prevents palmitate-induced insulin resistance by intrinsic PTP1B inhibition (Figure 7). Moreover, CYC31 also activates the fatty acid oxidation signaling in palmitate-exposed C2C12 myotubes. We believe that CYC31, a natural bromophenol PTP1B inhibitor, will provide a potential therapeutic candidate to prevent FFA-induced insulin resistance. However, it should be noted that this study has examined only cellular effects of CYC31; more research should be done in the future.

## 4. Materials and Methods

### 4.1. Materials

CYC31 was synthesized according to its natural structure in our lab [22]. Recombinant human PTP1B protein was also purified in our lab [18]. I-OMe AG538, insulin, palmitate, MTT, and fatty acid-free BSA were obtained from Sigma-Aldrich (St. Louis, MO, USA). 2-NBDG was obtained from Invitrogen (Carlsbad, CA, USA). DMEM, fetal bovine serum, and horse serum were bought from Hyclone (South Logan, UT). Penicillin-streptomycin solutions were purchased from Millipore (Billerica, MA, USA). ECL Western Blotting Substrates were bought from Bio-Rad (Hercules, CA, USA). pIRS1 (Tyr632) antibody and GLUT4 antibody were purchased from Santa Cruz Biotechnology (Dallas, TX, USA). pAkt (Ser473) antibody, pIRβ (Tyr1146) antibody, Insulin Receptor β antibody, and IRS1 antibody were bought from Cell Signaling Technology (Danvers, MA, USA). β-actin antibody was obtained from Abcam (Cambridge, MA, USA). RNAiso Plus, PrimerScript^TM^ RT reagent Kit, and SYBR Premix ExTaq^TM^ Ⅱ were obtained from Takara (Dalian, Liaoning, China).

### 4.2. PTP1B Enzymatic Assay

In vitro enzymatic activity of PTP1B was measured by using 4-nitrophenyl phosphate disodium salt (pNPP) as substrate. CYC31 and PTP1B_1-321_ protein was pre-incubated at room temperature for 5 min. Assay was conducted in a 96-well plate with reaction buffer containing Tris-Hcl (10 mM, pH 7.5), NaCl (25 mM), EDTA (1 mM), and pNPP (5 mM). The reaction mixture was then incubated at 37 °C for 30 min and stopped by adding 50 µL of 3 M NaOH. The absorbance density of *p*-nitrophenol was measured by a multi-well microplate reader at the wavelength of 405 nm.

### 4.3. Molecular Docking

The crystal structure of PTP1B (PDB ID: 3QKP) was obtained from the protein bank of RCSB. The three-dimensional structure of CYC31 was generated using Chembio3D Ultra 11.0, and then, the energy was minimized. AutoDock 4.0 program equipped with ADT was used to realize the automation of molecular docking. In Autodock calculation, using a grid spacing of 0.4 Å, a grid diagram of the catalytic site residues with WPD loop open conformation is defined for CYC31. The GA-LS algorithm adopts default settings. For docking operation, 200 GA-LS hybrid operations were conducted. A total of 200 possible binding conformations was generated and grouped based on 1.0 Å cluster tolerance. The docking model is analyzed and displayed by ADT.

### 4.4. Cell Culture and Viability Assay

C2C12 cells were bought from ATCC (Manassas, VA, USA) and cultured in DMEM supplemented with 10% FBS. Cell viability was evaluated by the MTT assay. Briefly, cells were plated into a 96-well plate at a density of 5 × 10^3^ cells/well. After overnight incubation, C2C12 cells in 10% horse serum was added to induce C2C12 cell differentiation for 4 days. Then, the cells were treated with 0.5–10 µM of CYC31 for 24 h. On the next day, cells were treated with 10 μL MTT (5 mg/mL) and subsequently incubated at 37 °C for 4 h. Finally, 150 µL of DMSO was added prior to reading. The absorbance density was determined using a multi-well microplate reader at the wavelength of 570 nm. Cells exposed to the DMSO alone were used as control.

### 4.5. Immunoblotting

For the insulin signaling assay, differentiated C2C12 cells were starved with serum-free DMEM overnight and then treated with CYC31 for 8 h to activate the insulin signaling pathway. For the insulin resistant assay, CYC31-treated cells were exposed to 0.5 mM palmitate-treated cells (PA) for 24 h and subsequently starved for another 6 h followed by 10 nM insulin treatment for 5 min.

Then, treated cells were washed by PBS and lysed with ice-cold RIPA buffer containing fresh PMSF. Total proteins were separated by SDS-PAGE and then transferred onto a PVDF membrane. Membranes were incubated with blocking buffer (5% skim milk in 1 × TBST) at room temperature for 1 h. Then, membranes were incubated with corresponding primary antibodies overnight at 4 °C. Then, HRP-linked secondary antibody was incubated with membranes at room temperature for 1 h. After 3-times washing with TBST, the bands were detected using ECL Substrate.

### 4.6. Measurements of mRNA

Total RNA was isolated with RNAiso Plus (Takara) according to the manufacturer’s instructions; 600 ng of total RNA was reverse transcribed to cDNA using M-MLV transcriptase (Takara) and oligo (dT) primer (Takara), respectively. cDNA was subsequently subjected to SYBR Green-based real-time PCR using an QuantStudio™ 6 Flex Real-Time PCR Systems (Applied Biosystems, Alameda, CA, USA). Primers used for amplification were as follows: Cpt-1, 5′-TTCACTGTGACCCCAGACGGG-3′ and 5′-AATGGACCAGCCCCATGGAGA-3′; 11β-HSD1, 5′-CCTTGGCTGGGAAAATGACC-3′ and 5′-CTATGAGGCCAAGGACACAGAGAG-3′; FABP3, 5′-CCCCTCAGCTCAGCACCAT-3′ and 5′-CAGAAAAATCCCAACCCAAGAAT-3′; IL-6, 5′-TCCAGCCAGTTGCCTTCTTGG-3′ and 5′-TCTGACAGTGCATCATCGCTG-3′; IL-10, 5′-GCCCTTTGCTATGGTGTCCTTTC-3′ and 5′-TCCCTGGTTTCTCTTCCCAAGAC-3′; β-actin, 5′-AGAGGGAAATCGTGCGTGAC-3′ and 5′-CAATAGTGATGACCTGGCGT-3′. The gene expression was normalized relative to the expression of the control gene (β-actin). The fold change of relative mRNA expression was calculated using the 2^−∆∆Ct^ method.

### 4.7. 2-NBDG Uptake

Cellular glucose uptake was determined according to the published procedure [32] by using a fluorescent 2-deoxyglucose analog, 2-(*N*-(7-nitrobenz-2-oxa-1, 3-diaxol-4-yl) amino)-2-deoxyglucose (2-NBDG) as probe. Briefly, C2C12 cells were plated in a black clear-bottomed 96-well culture plate with 1 × 10^4^ cells per well. After overnight incubation, the cells were induced to differentiation in DMEM containing 10% horse serum for 4 days. After overnight starvation, C2C12 myotubes were treated with the indicated concentrations of CYC31 for 8 h or 10 nM insulin for 5 min in serum-free DMEM. Then, 200 µM 2-NBDG was added for 20 min in glucose-free DMEM. At last, the cells were washed with PBS for three times, and the fluorescence intensity was measured at ex/em 465/540 nm by microplate fluorometer. Finally, the error caused by the difference of cell number was corrected by MTT method.

### 4.8. Membrane and Cytosol Protein Extraction

The Membrane and Cytosol Protein Extraction Kit (Beyotime, Shanghai, China) was used to extract the membrane and cytosol proteins. CYC31-treated C2C12 myotubes were washed with ice-cold PBS, and then, the membrane and cytosol proteins were fractioned according to the manufacturer’s instructions.

### 4.9. Statistical Analysis

Statistical significance was determined using one-way analysis of variance. Data were shown as means ± SD values. Data were considered significant at * *p* < 0.05, ** *p* < 0.01, and *** *p* < 0.001. SPSS 19.0 (SPSS, Inc., Chicago, IL, USA) was used for statistical analyses.
